# Effectiveness of Augmentative Biological Control of *Streptomyces griseorubens* UAE2 Depends on 1-Aminocyclopropane-1-Carboxylic Acid Deaminase Activity against *Neoscytalidium dimidiatum*

**DOI:** 10.3390/jof7110885

**Published:** 2021-10-20

**Authors:** Bader M. Al Hamad, Seham M. Al Raish, Gaber A. Ramadan, Esam Eldin Saeed, Shaikha S. A. Alameri, Salima S. Al Senaani, Synan F. AbuQamar, Khaled A. El-Tarabily

**Affiliations:** 1 Department of Biology, College of Science, United Arab Emirates University, Al Ain 15551, United Arab Emirates; 200337460@uaeu.ac.ae (B.M.A.H.); 200440261@uaeu.ac.ae (S.M.A.R.); jaber.ramadan@uaeu.ac.ae (G.A.R.); 201708673@uaeu.ac.ae (S.S.A.A.); 2 Hemaya Institute for Health, Safety, Environment and Food Science, Sharjah Research Technology and Innovation Park, Sharjah 66636, United Arab Emirates; 3 Khalifa Center for Genetic Engineering and Biotechnology, United Arab Emirates University, Al Ain 15551, United Arab Emirates; esameldin_saeed@uaeu.ac.ae (E.E.S.); s.alsenani@uaeu.ac.ae (S.S.A.S.); 4 Harry Butler Institute, Murdoch University, Murdoch, WA 6150, Australia

**Keywords:** ACC deaminase, actinobacteria, biological control agent, *Neoscytalidium dimidiatum*, stem canker, *Streptomyces*

## Abstract

To manage stem canker disease on royal poinciana, actinobacterial isolates were used as biological control agents (BCAs) based on their strong *in vitro* inhibitory effects against *Neoscytalidium* *dimidiatum*. *Streptomyces griseorubens* UAE2 and *Streptomyces wuyuanensis* UAE1 had the ability to produce antifungal compounds and cell-wall-degrading enzymes (CWDEs). Only *S. griseorubens*, however, restored the activity of 1-aminocyclopropane-1-carboxylate (ACC) deaminase (ACCD). *In vivo* apple fruit bioassay showed that lesion development was successfully constrained by either isolates on fruits inoculated with *N. dimidiatum*. In our greenhouse and container nursery experiments, *S. griseorubens* showed almost complete suppression of disease symptoms. This was evident when the preventive treatment of *S. griseorubens* significantly (*p* < 0.05) reduced the numbers of conidia of *N. dimidiatum* and defoliated leaves of royal poinciana seedlings to lesser levels than when *S. wuyuanensis* was applied, but comparable to control treatments (no pathogen). The disease management of stem canker was also associated with significant (*p* < 0.05) decreases in ACC levels in royal poinciana stems when *S. griseorubens* was applied compared to the non-ACCD-producing *S. wuyuanensis*. This study is the first to report the superiority of antagonistic actinobacteria to enhance their effectiveness as BCAs not only for producing antifungal metabolites and CWDEs but also for secreting ACCD.

## 1. Introduction

Plant pathogens can attack small ornamental shrubs or tall shaded trees [1,2]. *Neoscytalidium* species cause canker and dieback diseases on several woody hosts of agronomic, forestry, and ornamental importance [3,4,5,6]. For example, *Neoscytalidium dimidiatum* is the causal agent of stem canker disease on royal poinciana (*Delonix regia*) trees in the United Arab Emirates (UAE) [7]. This hardy beautiful tree has become a popular choice in the UAE due to its fast growth, nice shade and ability to adapt to different soil conditions [8]. Therefore, there is an urgent need to manage this destructive disease caused by *N. dimidiatum* in the UAE and elsewhere.

In modern agriculture, synthetic chemical fungicides no doubt provide protection against fungal pathogens [9,10,11]. Due to the impact of chemical fungicides on environmental and human health, natural compounds and other alternatives (e.g., horticultural practices) have recently been implemented in integrated disease management (IDM) strategies [12,13,14,15]. The native natural enemies of plant diseases, so-called biological control agents (BCAs), can be applied to effectively reduce disease development and yield loss of crops [16,17]. Previously, the bacterial isolates *Azotobacter chroococcum* and *Lactobacillus rhamnosus* [18] and the filamentous fungus *Trichoderma harzianum* [19] have been evaluated for their *in vitro* antagonism against *N. dimidiatum*.

Actinobacteria of the genus *Streptomyces* are major producers of bioactive compounds, antibiotics and extracellular enzymes for the agriculture, biotechnology and pharmaceutical industries [20]. *Streptomyces* spp. may have one or multiple mechanisms to control fungal pathogens [10,14] and to promote growth in plants [21,22]. For instance, pathogen growth can be inhibited by the production of cell-wall-degrading enzymes (CWDEs), antifungal metabolites or siderophores [23,24], hyperparasitism [25] and competition for nutrients [26]. Induced systemic resistance (ISR) is another resistance mechanism in plants that can be achieved by microbial inoculation [27].

A recent study by Al Raish et al. [28] has demonstrated that three actinobacteria, *Streptomyces rochei*, *S. coelicoflavus* and *S. antibioticus*, suppressed disease symptoms and prevent pathogen spread of *N. dimidiatum* under greenhouse conditions. The antifungal activities of *S. rochei* and *S. coelicoflavus* were associated with antibiosis and CWDEs production, respectively. *S. antibioticus* was, however, correlated with both mechanisms and had effectively capable to produce synergistic actions against the fungus under greenhouse conditions.

As highly abundant in many bacterial species, 1-aminocyclopropane-1-carboxylate (ACC) deaminase (ACCD) can help plant to grow under environmental stresses by decreasing the stress ethylene (ET) in plants [29,30]. ACCD breaks down ACC, an immediate precursor of ET, to ammonia (NH_3_) and α-ketobutyrate, which can be further used by bacteria for their growth [31,32]. Several studies have linked bacteria, including actinobacterial isolates, with the production of the inducible enzyme ACCD and plant growth promotion [32,33,34] and mitigation of abiotic stresses such as salinity and heavy metals pollution [30,32].

Except for a few studies in the past two decades, very little research in the field of plant disease management has been conducted on non-actinobacterial BCAs producing ACCD to enhance their antagonistic potential against phytopathogenic fungi [35,36,37,38]. To date, no attempts have been made to address the importance of ACCD in rhizosphere or endophytic BCAs belonging to actinobacteria, especially species from the genus *Streptomyces*. In the current study, we tested the hypothesis that the actinobacterial ACCD activity along with other modes of action act synergistically in the protection of royal poinciana seedlings against stem canker disease caused by *N. dimidiatum*. Accordingly, we screened a series of actinobacterial isolates for their efficacy as BCAs producing diffusible antifungal metabolites, volatile antifungal compounds, CWDEs, siderophores and ACCD on *N. dimidiatum* to protect royal poinciana orchards. Owing to ACCD activity, the preventive effect of *Streptomyces griseorubens* UAE2 helped royal poinciana seedlings to mitigate the effect of *N. dimidiatum* by decreasing *in planta* ACC and ultimately “stress ET” levels under container nursery conditions. Our results demonstrated that ACCD produced by *S. griseorubens* had additive protective effects as a BCA on diseased-royal poinciana compared to other actinobacterial isolates that did not have this feature; albeit all other beneficial mechanisms. Thus, *S. griseorubens* is an antagonist that can be potentially associated with IDM programs to manage stem canker disease on royal poinciana.

## 2. Materials and Methods

### 2.1. Fungal Culture and Isolation of Actinobacteria from Soil Samples

In this study, the fungal pathogen *N. dimidiatum* (DSM 109897) [7] was cultured on potato dextrose agar (PDA; Lab M Limited, Lancashire, United Kingdom) plates. Every 10 days, the growing fungus was subcultured on PDA plates at 28 °C.

To isolate actinobacteria, six rhizosphere soil samples were collected from under healthy royal poinciana trees (depth of 30 cm) in Dubai, UAE. The rhizosphere soils were air-dried for 4 days at 28 °C to reduce the numbers of viable vegetative bacterial cells [39].

For streptomycete actinobacteria (SA) isolation, the soil dilution plate method [40] using inorganic salt starch agar (ISSA) [41] with specific soil pre-treatments [42] was used. Briefly, the soil was pre-treated when serial dilutions of the soil suspension were prepared by suspending the sample in yeast extract (YE) (Sigma–Aldrich Chemie GmbH, Taufkirchen, Germany) and sodium dodecyl sulfate (SDS) (Sigma–Aldrich) (6% YE + 0.5% SDS) for 20 min at 40 °C and diluting with water to remove other factors promoting bacterial growth or injurious to germinating actinobacterial spores [43]. The YE and SDS were added to decrease and increase the populations of bacteria and actinobacteria, respectively [44].

Two techniques were also employed to isolate non-streptomycete actinobacteria (NSA) from the rhizosphere: (i) the application of polyvalent *Streptomyces* phage [45]; and (ii) the use of dry heat method [43]. These methods reduced SA dominance on isolation plates in order to facilitate the recovery of NSA from rhizosphere soils.

For the phage method, two polyvalent *Streptomyces* phages [23] were used. The stock phage suspension was prepared by combining high-titer suspensions (×10^12^ plaque forming units/mL) of each phage, and the stock suspension was then used to treat 10 g of soil suspensions (five replicates) in dilution tubes. Actinobacterial colonies were isolated on ISSA agar plates. ISSA plates were dried in a laminar flow cabinet for 20 min and incubated at 28 °C in dark for two weeks. Plates inoculated with soil dilutions not treated with the polyvalent phages were used as control treatments.

For the dry heat method, five replicates of soil (10 g each) were heated at 120 °C for 1 h. Heat-treated soil (1 g) was added to 10 mL of sterile distilled water in a test tube and vortexed for 5 min. Further dilutions (10^−2^, 10^−3^ and 10^−4^) were prepared in sterile distilled water. Control treatments consisted of 1 g of unheated soils. Colonies of actinobacterial were isolated using the spread plate technique over arginine vitamin agar (AVA) [43]. AVA plates were dried in a laminar flow cabinet for 20 min and were incubated at 28° in the dark for two weeks to encourage NSA growth. In the two media used (ISSA and AVA), the antifungal antibiotics, cycloheximide and nystatin (each 50 µg/mL; Sigma–Aldrich) were added to the cooled (45 °C) sterile media immediately prior to pouring plates.

SA and NSA colonies were counted [log_10_ colony forming units (cfu)/g dry soil] and purified on oatmeal agar plates (ISP-3 medium) amended with 0.1% yeast extract (OMYEA) [41], stored and tentatively identified according to Cross [46]. Differentiation between SA and NSA colonies was based on morphological criteria and the presence or absence of aerial mycelium, distribution (aerial/substrate), form of spores and stability of substrate mycelium.

### 2.2. In Vitro Screening for Diffusible Antifungal and CWDE Activities

Antifungal activities of all isolates were characterized based on the secretion of diffusible antifungal metabolites against *N. dimidiatum* using the cut-plug method [47]. Actinobacterial isolates were inoculated on fish meal extract agar (FMEA) plates and incubated at 28 °C in the dark for seven days [10,25]. Briefly, plugs from the actinobacterial cultures growing on FMEA were transferred on PDA plates seeded with *N. dimidiatum* using a sterilized 11-mm cork-borer and kept at 28 °C in the dark for five days. The diameters of the inhibition zone were determined. Eight plates/actinobacterial isolate were used. Isolates showing a large inhibition zone (>25 mm) on plates were further considered; whereas the rest of the isolates were discarded.

For preliminary assessment of CWDEs production, all isolates were tested for their abilities to produce clearing zones on *N. dimidiatum* mycelial fragment agar (MFA) [48]. Similarly, high CWDE activities associated with large diameters of clearing zones (>25 mm) were considered; whereas the remaining were not further selected. In addition, all obtained isolates were evaluated for the production of the chitinase enzyme. Each isolate was inoculated onto colloidal chitin agar (CCA) plates, and incubated at 28 °C in the dark for seven days [49]. The clearing zones surrounding the colonies were measured, where diameters of >25 mm and <25 mm represented high and low chitinase activities, respectively. Eight plates/isolate were used.

Only the most promising diffusible antifungal metabolite-producers on FMEA and the highly active CWDE-producing isolates on MFA and CCA plates were chosen for further analyses.

### 2.3. In Vivo Apple Fruit Bioassay

To determine whether the candidate isolates can reduce lesion formation of *N. dimidiatum*, an *in vivo* apple fruit bioassay was performed [7]. Mature apple fruits (cv. Granny Smith) placed in plastic trays on sterile paper towels moistened with sterile distilled water were inoculated with agar plugs (11 mm diameter) colonized by isolates and/or *N. dimidiatum* onto each apple fruit as the following: (1) two sterile, non-inoculated PDA agar plugs on each other (control; C); (2) the antagonist alone (BCA) with a sterile PDA agar plug on top of it; (3) *N. dimidiatum* (*Nd*) alone with a sterile PDA agar plug underneath it; and (4) pairing *N. dimidiatum* and the antagonists together (BCA + *Nd*), with the BCA on the apple surface and *N. dimidiatum*-inoculated plug on top of the BCA. The antagonists were inoculated onto the apple surface 24 h prior the pathogen to give the actinobacterial isolate the time to secret antifungal metabolites and/or CWDEs onto the fruit. Trays were covered with aluminum foil to maintain humidity at 28 °C for five days and lesion diameters were measured. All diseased fruits were incubated on PDA plates at 28 °C in the dark for five days to fulfill Koch’s postulates. The four combinations were applied on each fruit, where five fruits/tray were randomly selected and evaluated.

Only the seven promising diffusible antifungal metabolite-producers and the highly active CWDE-producing isolates that completely prevented lesion formation on apple fruits were chosen for subsequent experiments.

### 2.4. Qualitative and Quantitative Determination of ACCD Activity by Actinobacterial Isolates

The aim of this experiment was to screen all seven BCAs from the previous experiment for their potential to produce ACCD from ACC using the nitrogen-free Dworkin and Foster’s salts minimal agar medium (DF) [50]. The medium was supplemented with either 2 g/L (NH_4_)_2_SO_4_ or 3 mM ACC (Sigma–Aldrich) as a sole nitrogen source. The heat-labile ACC was sterilized through sterile Millipore membranes (pore size 0.22 μm, Millipore Corporation, MA, USA) and the filtrate was added to the salt medium after autoclaving.

Five-day-old isolates grown on rich OMYEA were streaked in triplicate on DF agar medium plates amended with either (NH_4_)_2_SO_4_ or ACC. The plates were incubated at 28 ± 2 °C in the dark for seven days. Growth and heavy sporulation of the isolates on DF agar medium amended with ACC (DF-ACC agar) was taken as an indicator of the efficiency of selected isolates to utilize ACC and to produce ACCD.

For the quantitative determination of ACCD activity, BCA isolates were grown in inorganic salt starch broth (ISSB) [41] at 28 ± 2 °C in the dark for five days. Spores were harvested by centrifugation, washed twice with 0.1 M Tris-HCl (pH 8.5) and inoculated onto DF-ACC broth. Flasks were incubated on a rotary shaker (Model G76, New Brunswick Scientific, NJ, USA) at 250 rpm at 28 ± 2 °C in the dark for five days. Cells were collected, resuspended in 0.1 M Tris-HCl, and ruptured by three freeze-thaw cycles (immersion in liquid nitrogen for 1 min, followed by 5 min in water bath at 25 °C [51]. The lysate was then centrifuged at 80,000× *g* for 1 h and the supernatant was quantified for ACCD activity by monitoring the amount of α keto-butyrate produced by the deamination of ACC [52]. Protein concentrations were determined using a Bradford assay [53]. Eight independent replicate flasks for each isolate were analyzed.

### 2.5. Production of Volatile Antifungal Compounds, Siderophores and Hydrogen Cyanide

The promising seven BCAs were also tested on FMEA for the production of volatile antifungal compounds [54]. For the production of siderophore, plates of chrome azurol S (CAS) agar developed by Schwyn and Neilands [55], were inoculated with the concerned BCAs and incubated at 28 °C in dark for seven days. Development of yellow-orange halo zone around the colony represents a siderophore-producing isolate.

For the production of hydrogen cyanide (HCN) which should be followed with extreme caution, plates of tryptic soy agar medium (Lab M Limited) supplemented with 4.4 g/L glycine were inoculated with the BCAs. Plates were inverted and a piece of filter paper, soaked in 0.5% picric acid +2% sodium carbonate, was placed in the lid of each Petri dish, and incubated at 28 °C for five days [56]. After incubation, discoloration of the filter paper to orange brown indicates HCN production [57].

### 2.6. Greenhouse Experiments

To choose the most effective BCAs against *N. dimidiatum*, the seven potential isolates were tested prior to inoculation with *N. dimidiatum* on royal poinciana seedlings in the greenhouse. It is noteworthy to mention that some of these BCAs were found to be ACCD producers and others were non-ACCD producers.

A pathogenicity test was conducted on one-year-old healthy royal poinciana seedlings. Under-bark inoculation was performed at 30–35 cm above the root collar using a sterile scalpel to wound the bark, and placing an agar plug (8 mm diameter) colonized by mycelium of a 10-day-old culture of *N. dimidiatum* into the wound with mycelium facing the stem [7]. Each wound was wrapped in parafilm to maintain moist conditions. Seedlings of royal poinciana were inoculated with sterile agar plugs as negative controls. Plants were monitored for symptoms at four weeks post inoculation (wpi). The fungus was re-isolated from the infected stems on PDA at the end of the experiment and morphologically compared with the inoculated fungus.

Similar to the pathogenicity test described above, methods of pathogen inoculation and BCA application were used. For BCA treatments, seedlings were treated with each BCA for one week before *N. dimidiatum* inoculation in order to ensure stem colonization by that particular BCA.

For each treatment/group, eight separate pots each containing one seedling were arranged in a completely randomized design. Control and inoculated seedlings were maintained in the greenhouse (15 h day/9 h night; 160 µmol/m^2^/s fluorescent lights; 28 ± 2 °C). To determine disease symptoms/recovery of inoculated plants, conidial counts of the fungal pathogen and the number of falling leaves were recorded at five weeks post treatment (wpt) [7]. Conidia were harvested from affected tissues (three leaf bases of eight inoculated seedlings per treatment) in 5 mL of water, and counted using haemocytometer (Agar Scientific Limited, Essex, UK).

### 2.7. Production of Antifungal Compounds and CWDEs

From the data obtained in Section 2.6, only the two strongest BCAs, BCA3 (the ACCD producer) and BCA5 (the non-ACCD producer) were chosen for further detailed studies below. The cup plate technique [58] was also used to assess secretion of diffusible antifungal metabolites by the selected antagonistic BCAs against *N. dimidiatum*. The diameter of inhibition zones was determined for the two selected BCA after five days of incubation in the dark at 28 °C.

To evaluate the inhibition of *N. dimidiatum* by the diffusible antifungal metabolites on FMEA or by the CWDEs on CCA, a dialysis membrane overlay technique [59] was performed. The dialysis membrane (Type 45311; Union Carbide Corporation, IL, USA) with adhering BCA colonies was removed from agar plates and the center of each plate was inoculated with a disc (5 mm diameter) of *N. dimidiatum* culture. After incubation, colony diameters of *N. dimidiatum* were measured. The agar plugs were further transferred to a fresh PDA plate and incubated at 28 °C for five days to determine if the diffused metabolites/chitinase were fungistatic (pathogen growing from plug) or fungicidal (no pathogen growing from plug).

### 2.8. Quantitative Production of CWDEs by BCAs

Two mg/mL of either *N. dimidiatum* cell-wall fragments, colloidal chitin or laminarin (Sigma–Aldrich) were placed in flasks containing 50 mL of minimal synthetic medium [60]. Each substrate in flasks was inoculated with 2 mL of a 20% glycerol suspension of each BCA (10^8^ cfu/mL), placed on a rotary shaker (New Brunswick Scientific) for seven days at 250 rpm speed, followed by 12,000× *g* centrifugation for 30 min. The supernatant, which was used as a source of crude enzymes [61] was filtered using 0.22 µm Millipore membranes (Millipore Corporation). The release of N-acetyl-D-glucosamine and the amount of reducing sugars liberated using dinitrosalicylic acid solution [62] determined the activities of chitinase and β-1,3-glucanase, respectively. The protein content in the enzyme solution was determined using Folin phenol reagent as described by Lowry et al. [63].

### 2.9. Effect of Crude Culture Filtrates of BCAs on N. dimidiatum

For the two promising BCAs, filter-sterilized crude culture filtrate (described in Section 2.8) using fish meal extract broth (FMEB) or colloidal chitin broth (CCB) [49] was proportionally poured in PDA plates. The medium was inoculated with an agar plug (5 mm diameter) colonized with *N. dimidiatum* mycelium by placing it upside down. The colony diameter (mm) of the pathogen was measured after five days at 28 °C. In addition, the prepared crude culture filtrate from FMEB or CCB was proportionally mixed with potato dextrose broth (PDB; Lab M) [64]. Similarly, PDB was inoculated with a 5-mm diameter agar plug of the pathogen; and *N. dimidiatum* dry weight was measured after 10 days of incubation in the dark at 28 °C.

We also tested the effect of the crude culture filtrate of the BCAs on mature conidia germination and germ tube elongation of *N. dimidiatum* on PDB [64]. We microscopically determined and compared the percentage of spore germination and the average of germ tube length using Nikon-Eclipse 50i light microscope at 40× with a total magnification of 400× after 24 h (Nikon Instrument Inc., Melville, NY, USA) with the non-inoculated control FMEB or CCB.

The crude culture filtrate of BCAs was also assessed on the hyphal morphology of *N. dimidiatum* [65] using oil immersion lens (100×) with a total magnification of 1000×. Control treatments of *N. dimidiatum* mycelium incorporated with non-inoculated filter-sterilized FMEB or CCB were also investigated.

### 2.10. Cultural, Morphological and Molecular Identification of the BCA

Two promising BCAs were identified based on their 16S rRNA gene sequence analysis using the primers 900R (5′-CCGTCAATTCATTTGAGTTT-3′); 357F (5′-TACGGGAGGCAGCAG-3′) and 800F (5′-ATTAGATACCCTGGTAG-3′) [66] done by Deutsche Sammlung von Mikroorganismen und Zellkulturen GmbH, (DSMZ), Braunschweig, Germany. All sequences were deposited in Genbank with accession numbers MZ021577 for BCA3 (isolate #21) and MZ026483 for BCA5 (#45). The 16S rRNA gene similarity values were calculated by pairwise comparison of the sequences within the alignment. To construct species-level phylogenies, the tree was based on Maximum Likelihood method and Tamura-Nei model [67] implemented in Molecular Evolutionary Genetics Analysis version X (MEGAX) software [68] and were applied after data alignments by CLUSTAL_X [69]. In each case, bootstrap values were calculated based on 500 resamplings.

Cultural, morphological, physiological and biochemical characteristics of BCA isolates were also determined [70]. Spore morphology for each promising BCA isolate was observed using scanning electron microscopy (SEM) of XL-30 from Philips (FEI Co., Eindhoven, The Netherlands).

### 2.11. Container Nursery Experiments

In the nursery experiment, the strongest BCAs were evaluated on royal poinciana seedlings. Our aim was to test the efficacy of BCAs prior infection with *N. dimidiatum* on royal poinciana. Except for the extended inoculation time point using the individual BCAs, pathogenicity tests and inoculation methods with the pathogen and BCA were described in Section 2.6. Seedlings were treated with each BCA for two weeks before *N. dimidiatum* infection to ensure stem colonization by that particular BCA.

In this experiment, the five treatments were as follows:

(i) Healthy controls (C): Non-inoculated control seedlings;
(ii) Diseased controls (*Nd*): Seedlings inoculated with *N. dimidiatum* only;
(iii) Isolate #9 treatment: Seedlings inoculated with the non-BCA, ACCD-producing isolate #9 followed by *N. dimidiatum* inoculation (positive control);
(iv) BCA3 treatment: Seedlings inoculated with the BCA and ACCD-producing isolate #21 followed by *N. dimidiatum* inoculation; and
(v) BCA5: Seedlings inoculated with the BCA and non-ACCD-producing isolate #45 followed by *N. dimidiatum* inoculation.

In treatments where actinobacterial isolates were used, seedlings were inoculated with the isolate for two weeks followed by *N. dimidiatum* inoculation for another eight weeks (when leaves of control seedlings were completely defoliated).

For each treatment, eight separate pots each containing one seedling were arranged in a completely randomized design. The container nursery experiments were independently repeated twice. Control and inoculated seedlings were maintained under natural conditions between February to April (relative humidity range = 24–41%; daytime length range = 8.5–9.5 h day; average temperature = 29 ± 4 °C day/16 ± 3 °C night; average precipitation = 13.3 mm). To determine disease symptoms/recovery of inoculated plants, conidial counts of the fungal pathogen and the number of falling leaves were recorded at 10 wpt as described in Section 2.6.

### 2.12. Extraction and Measurement of Endogenous ACC from Stem Tissues

Determination of endogenous ACC contents was carried out at the end of the natural container nursery experiment from stems according to Lizada and Yang [71]. Stem pieces (each piece was 10 cm in length including the inoculation point ± 5 cm below and above that point) were collected.

Derivatization of ACC was carried out by adding phenylisothiocyanate (Sigma) [72]. HPLC chromatograms were produced by injecting 10 µL of the resulting phenylthiocarbamyl-ACC samples dissolved in acetonitrile onto a 10-µm reverse phase column (Waters Associates µBondapak C_18_, 4 mm × 30 cm) in a Waters Associates liquid chromatograph equipped with a differential UV detector set at 254 nm as described by Lanneluc-Sanson et al. [72]. Eight independent replicate samples were analyzed.

The ACC concentrations were obtained by comparing their respective peak areas in the unknown sample with their corresponding areas obtained with the authentic samples (Sigma–Aldrich) of a known concentration.

### 2.13. Statistical Analyses

All *in vitro* experiments were independently repeated eight times. Similar results were obtained in each replicate. For *in vitro* evaluation of BCA against *N. dimidiatum*, Analysis of Variance (ANOVA) and Duncan’s multiple range test at *p* = 0.05 were used on eight plates/treatment. For apple fruit bioassays, each fruit was inoculated with the four treatments and each tray (five trays were used) containing five fruits. The effect of BCAs on lesion formation was analyzed using ANOVA, and significant differences between means at *p* = 0.05 were determined by Duncan’s multiple range test.

All greenhouse and container nursery experiments were independently repeated twice with similar results, and the obtained data were combined and analyzed. For the falling leaves and fungal conidia counts of the *in vivo* treatments against *N. dimidiatum*, 16 plants (eight replicates from each experiment) were used for each treatment. ANOVA and Duncan’s multiple range test were used to determine the statistical significance (*p* < 0.05). For all statistical analyses, SAS Software version 9 was used (SAS Institute Inc., Cary, NC, USA).

## 3. Results

### 3.1. In Vitro Production of Diffusible Antifungal Metabolites and CWDEs by SA and NSA

In our efforts to isolate actinobacteria from royal poinciana rhizosphere, a total of 47 strains of which 38 SA (80.8%) and 9 NSA (19.1%) were successfully obtained on ISSA plates. To increase the diversity of NSA isolates, two techniques (polyvalent *Streptomyces* phages and dry heat) were also used.

When *Streptomyces* phages with high polyvalency were used, numbers of NSA significantly (*p* < 0.05) increased by 33.1% (Table S1). Dry heat treatment of soils significantly (*p* < 0.05) reduced SA numbers compared to untreated soils, but significantly increased NSA numbers by 31.8% (Table S1). An additional 21 and 23 NSA isolates were obtained after using the phages and dry heat methods, respectively.

A total of 91 isolates (47 SA and 44 NSA) were identified to the genus level according to their morphology, presence of aerial mycelia, and the stability of substrate mycelia and distribution of aerial and substrate mycelia. *Actinomadura*, *Actinoplanes*, *Dactylosporangium*, *Streptosporangium*, *Micromonospora* and *Microbispora* spp. were among the NSA strains that were isolated (Table 1).

Two *in vitro* experiments were conducted in parallel to screen for the production of both diffusible antifungal metabolites and CWDEs. Our results showed that 12 SA (out of 47) and eight NSA (out of 44) isolates displayed strong production of both diffusible antifungal metabolites and CWDEs against *N. dimidiatum* (Table 1; Figure S1). Together, the 20 isolates producing large zones of pathogen inhibition (>25 mm) on FMEA as well as large clearing zones (>25 mm) on MFA were considered as promising BCA candidates. SA isolates #4, #8, #10, #19, #21, #23, #29, #32, #37, #44, #45 and #53 and NSA isolates #7, #14, #25, #38, #41, #42, #48 and #56 (Table 1) were selected for further analysis. The rest of the isolates showing <25 mm of inhibition zones and/or clearing zones were not included in subsequent studies. This suggests that the identified SA and NSA that were isolated from royal poinciana rhizosphere soils found in the UAE could be potentially effective on *N. dimidiatum*.

### 3.2. In Vivo Selection of the Most Likely BCA Candidates to N. dimidiatum

**Figure 1:**
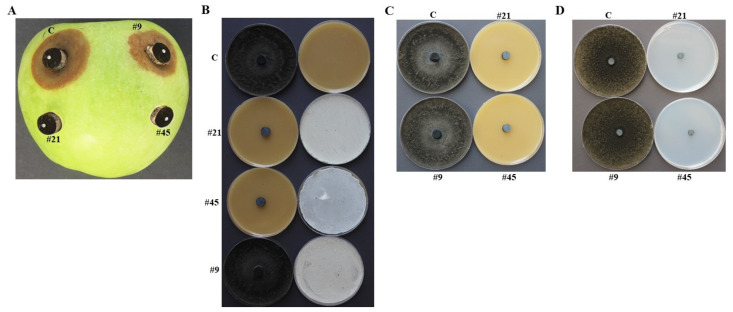
*In vivo* apple bioassay and *in vitro* characteristics of BCA candidates against *Neoscytalidium dimidiatum*. (**A**) Apple fruit bioassay using agar plug combinations of inoculated-apple fruit with BCAs and *N. dimidiatum* after seven dpi. (**B**) Inhibition of *N. dimidiatum* mycelial growth by the volatile antifungal compounds-producing isolates #21 (BCA3) and #45 (BCA5) compared to the volatile antifungal compounds-non-producing isolate #9. Dialysis membrane overlay technique using (**C**) FMEA or (**D**) CCA plates. In (**A**), *N. dimidiatum* inoculum alone with a sterile agar plug below it (**C**); isolate #, the isolate was placed on the apple surface and *N. dimidiatum*-inoculated plug on top of the isolate. In (**C**), inhibition of *N. dimidiatum* mycelial growth was observed on FMEA plates colonized by the diffusible antifungal metabolite-producing isolate #21 (BCA3) and #45 (BCA5) compared to the diffusible antifungal metabolite-non-producing isolate #9. In (**D**), inhibition of *N. dimidiatum* mycelial growth was observed on CCA plates colonized by the chitinase-producing isolates #21 and #45 compared to the chitinase non-producing isolate #9. In (**B**–**D**), no isolate colonization served as a control (**C**). In (**A**–**D**), the diffusible antifungal metabolite- and CWDEs-producing isolates #21 and #45 were compared to the non-producing isolate #9. Isolates #21, #45 and #9 represent *Streptomyces griseorubens* (BCA3), *Streptomyces wuyuanensis* (BCA5) and *Streptomyces* sp.; respectively. FMEA, fish meal extract agar; CCA, colloidal chitin agar, dpi, days post inoculation; BCA, biological control agent.

An apple fruit bioassay method was used to check the efficacy of the 20 BCA candidates against *N. dimidiatum* (Table 1). We observed relatively large brownish lesions on apple fruits that were produced by the pathogen (*N. dimidiatum*) alone or with the non-antagonistic positive control *Streptomyces* sp. isolate #9 (Figure 1A). Except of those that almost completely prevented disease lesion formation on the surface of apple fruits, the rest were excluded. For that reason, only five SA (#4, #10, #21, #23 and #45) and two NSA (#38 and #42) isolates paired with the pathogen on apple fruits were considered (Table 1; Figure 1A).

Because we aimed to find actinobacterial isolates possessing not only BCA properties, but also enzymatic ACCD activities, we grew the potential BCAs on DF plates amended with ACC. Activities of ACCD significantly (*p* < 0.05) varied among isolates. Three isolates, #4 (BCA1), #38 (BCA7) and #21 (BCA3) were regarded as low, medium and high ACCD producers; respectively. On the other hand, four isolates #10 (BCA2), #23 (BCA4), #42 (BCA6) and #45 (BCA5) were considered as non-ACCD producers (Table 1). These 7 isolates were further tested as BCAs and/or ACCD-producing isolates under greenhouse conditions.

Except of BCA1 and BCA6, the other BCAs produced volatile antifungal compounds and suppressed pathogen growth *in vitro* (Table S2; Figure 1B). Although five of the BCAs produced siderophores, BCA2 and BCA7 failed to do the same (Table S2). After following extreme caution, all the seven BCAs failed to produce HCN (Table S2).

### 3.3. Evaluation of Potential BCA Candidates on N. dimidiatum in the Greenhouse

**Figure 2:**
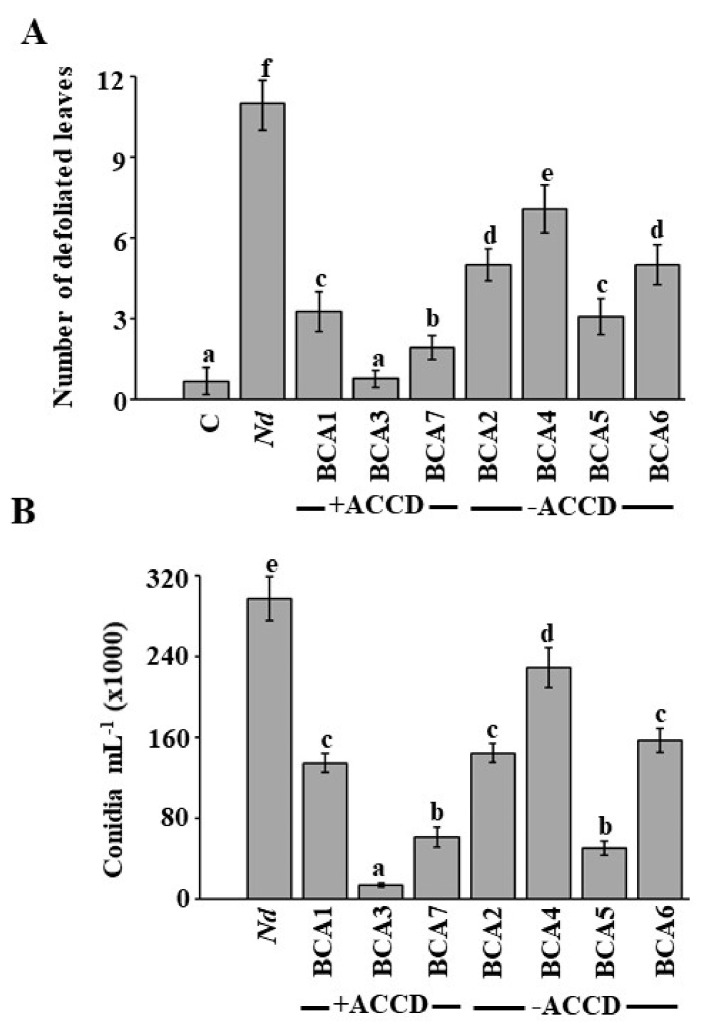
Effect of the application of selected *Streptomyces* spp. on royal poinciana stem canker disease under greenhouse conditions. Preliminary results of BCA treatments on (**A**) number of defoliated leaves of inoculated seedlings; and number of (**B**) conidia after the recovery of the pathogen from affected stem tissues after five wpt. In (**A**,**B**), values with different letters are significantly different from each other at *p* < 0.05. Bars represent standard errors. Royal poinciana seedlings were inoculated with the individual isolate one week prior to *Neoscytalidium dimidiatum* inoculation. BCA1, BCA3 and BCA7 are ACCD-producing isolates (+ACCD), whereas BCA2, BCA4, BCA5 and BCA6 are ACCD-non-producing isolates (−ACCD). C, non-inoculated control seedlings, *Nd*, seedlings inoculated with *N. dimidiatum* only; BCA, biological control agent; ACCD, 1-aminocyclopropane-1-carboxylic acid deaminase; wpt, weeks post treatment. Isolates #21 (BCA3) and #45 (BCA5) represent *Streptomyces griseorubens* UAE2 and *Streptomyces wuyuanensis* UAE1, respectively.

To test the efficacy of the seven potential BCAs, a greenhouse experiment on stem canker diseased royal poinciana was carried out. Disease symptoms such as the number of falling leaves and the recovery of *N. dimidiatum* on seedlings (*Nd*) as disease severity/recovery indices with or without BCA treatments were determined. There was a significant (*p* < 0.05) difference in the number of defoliated leaves among BCA treatments on diseased-seedlings (Figure 2A). After applying BCA3 on diseased seedlings at five wpt (corresponding to four wpi with *N. dimidiatum*), we did not observe major differences in responses of plants compared with control (no pathogen) treatment (Figure 2A). Thus, responses of seedlings recovered at the end of the experiment were completely the opposite in the seedlings inoculated with *N. dimidiatum* only. Plants treated with either BCA1 or BCA7, representing two other BCAs producing ACCD, one-week before inoculation with *N. dimidiatum*, reduced the number of defoliated leaves resulting from infection to lesser levels than those in plants treated with BCA3. Following inoculation with the pathogen, plants inoculated with any of the non-ACCD-producing isolates also showed relatively healthy plants (Figure 2A) when compared to seedlings inoculated with *N. dimidiatum* only at the tested time point. Thus, BCA5 treatment was the best treatment among all the ACCD-non-producing isolates, but was comparable to BCA1 treatment. These results suggest that ACCD-producing isolates may have a major effect on reduction of falling leaves, thereby relieving plants from biotic stresses i.e., *N. dimidiatum*.

We also determined pathogen responses to all BCA treatments on the number of conidia recovered from treated seedlings. Similar to what we observed with the low number of falling leaves (Figure 2A), the preventive application of BCA3 dramatically reduced the number of conidia, followed by both seedlings treated with BCA7 and BCA5 (Figure 2B). There was no significant (*p* > 0.05) difference in total number of conidia of *N. dimidiatum* among individual treatments of BCA1, BCA2 and BCA6, thus BCA4 was the least efficient (Figure 2B). In these trials, none of the preventive treatments of BCAs reached to level of protection as found by the BCA3 isolate.

In general, diseased plants inoculated with BCA3 (the highly active ACCD-producing isolate) looked healthy with slight effect of *N. dimidiatum* on seedlings. Our data imply that preventive treatments of BCAs possessing more than one mechanism of action may not be sufficient to successfully manage stem canker disease on royal poinciana, thus ACCD secretion by the BCA can be the key player in inhibiting pathogen growth *in planta*.

### 3.4. In Vitro Comparisons of Antagonistic Activities between BCA3 and BCA5

We also carried out an additional *in vitro* screening to examine the exact mode of action(s) to distinguish between BCA3 and BCA5 apart from ACCD enzymatic activity. By using the cup plate (Table S3; Figure S1) and dialysis membrane from FMEA (Figure 1C) methods, the *in vitro* effect of the culture filtrate of the two selected BCAs on *N. dimidiatum* growth was not significant (*p* > 0.05). This suggests almost similar effective diffused antifungal metabolites in BCA3 and BCA5 was found. *N. dimidiatum* did not recover from plugs when transferred from treated plates to fresh PDA, confirming that the metabolites diffused by BCA3 and BCA5 were fungicidal.

Similar quantitative *in vitro* results were obtained with the dialysis membranes from CCA plates of BCA3 and BCA5 that inhibited growth of *N. dimidiatum* (Figure 1D). The failure of *N. dimidiatum* to grow from plugs transferred to new PDA when the diffused CWDEs were absent indicated that BCA3 and BCA5 showed fungicidal activities. The production of chitinase by the two BCAs on the amended media with colloidal chitin or *N. dimidiatum* cell-walls was insignificant (*p >* 0.05; Table S3). Both BCAs produced high levels of β-1,3-glucanase. This was evident in the β-1,3-glucanase detected by BCA3 and BCA5 growing in media containing laminarin or *N. dimidiatum* cell-walls (Table S3), thus insignificant (*p >* 0.05) from each other.

### 3.5. Effect of Crude Antifungal Culture Substances of BCA Candidates on N. dimidiatum

As the concentration of filter-sterilized crude culture filtrates of BCA3 and BCA5 increased in FMEB, both colony diameter and mycelial dry weight of *N. dimidiatum* were significantly (*p* < 0.05) affected (Table 2). Thus, they were completely inhibited on PDA plates when crude culture filtrates reached to 100%. Similarly, crude culture filtrates of BCA3 and BCA5 from CCB significantly (*p* < 0.05) decreased colony and mycelial growth of *N. dimidiatum* when proportionally added into PDB.

In addition, conidial germination and the average germ tube length of *N. dimidiatum* were significantly (*p* < 0.05) reduced when the crude culture filtrate of the two BCA candidates were collected from FMEB and CCB (Table 2). This suggests that the increasing levels of crude culture filtrates of BCA3 and BCA5 were effective against different components (e.g., mycelia and conidia) of *N. dimidiatum*, thus inhibiting the fungal growth.

**Figure 3:**
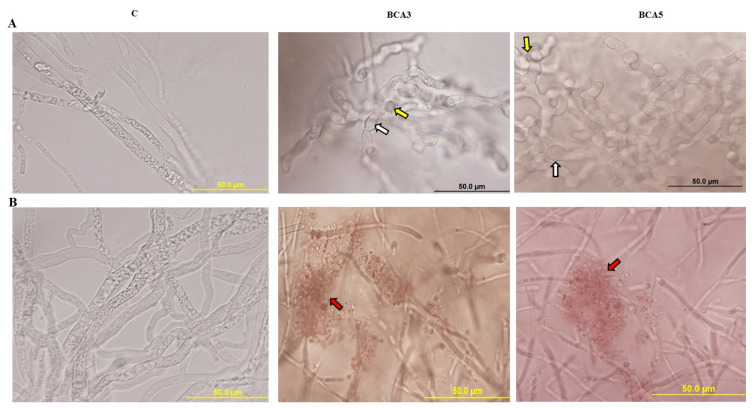
Effect of the culture filtrate of the BCA candidates on hyphae and cytoplasm of *Neoscytalidium dimidiatum.* Abnormalities observed in hyphal morphology and cytoplasmic contents of *N. dimidiatum,* following exposure to a filter-sterilized crude culture filtrate of BCAs on (**A**) FMEB, or (**B**) CCB compared to control. White and yellow arrows point to hyphal septum malformation and branch deformation, and cytoplasmic coagulation, respectively. Red arrows point to lysis of cytoplasm. Light microscopy images were taken at 1000× magnification. C, control (no BCA was applied). Isolates #21 (BCA3) and #45 (BCA5) represent *Streptomyces griseorubens* UAE2 and *Streptomyces wuyuanensis* UAE1, respectively. FMEB, fish meal extract broth; CCB, colloidal chitin broth; BCA, biological control agent.

We also tested the crude culture filtrate of BCA candidates from FMEB and CCB on hyphae and cytoplasm of *N. dimidiatum*. We observed hyphal swelling and cytoplasmic coagulation of *N. dimidiatum* when exposed to the crude culture filtrate of BCA3 and BCA5 from FMEB (Figure 3A). Culture filtrates of the same BCAs obtained from CCB illustrated hyphal lysis of the pathogen (Figure 3B). Mycelial mats produced by the fungal pathogen were intact in control treatments.

Together, our data confirm that BCA3 and BCA5 can cause relatively similar damage to *N. dimidiatum* through the production of antifungal metabolites and CWDEs. Hence, a comparison between BCA candidates would demonstrate if there were any additive effect(s) of the ACCD-producing BCA3 on *N. dimidiatum* inoculated-royal poinciana in the greenhouse.

### 3.6. Identification and Phylogeny of the Candidate BCA Species

**Figure 4:**
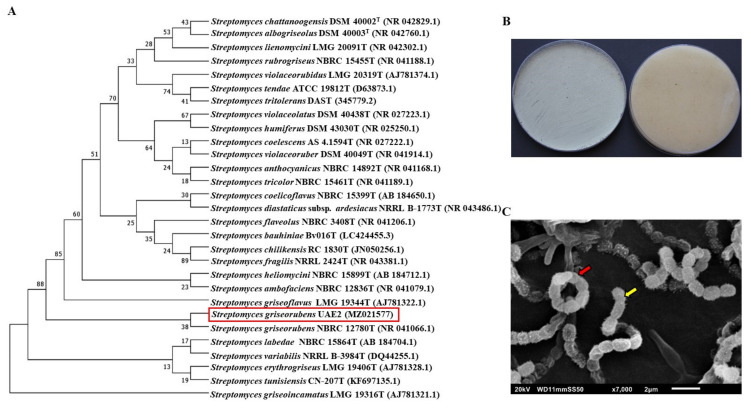
Phylogenetic, cultural and morphological characteristics of *Streptomyces griseorubens* UAE2 (BCA3, ACCD-producing isolate). (**A**) The phylogenetic tree showing the relationships between *S. griseorubens* UAE2 (isolate 21; MZ021577; 1518 bp) and other *Streptomyces* spp. according to 16S rRNA sequences. (**B**) Gray aerial (left) and colorless substrate (right) mycelia growing on ISP3 medium supplemented with yeast extract. (**C**) Scanning electron micrograph (7000×) of spiral spore chains (red arrow) and spiny-surfaced spores (yellow arrow) of *S. griseorubens* UAE2. In (**A**), numbers at nodes indicate percentage levels of bootstrap support based on a neighbor-joining analysis of 500 resampled datasets. GenBank accession numbers are given in parentheses. BCA, biological control agent.

The two antagonists, BCA3 and BCA5, were identified based on 16S rRNA gene sequence with other *Streptomyces* spp. The 1518-bp sequence of BCA3 (#21), designated a GenBank accession number MZ021577, showed 100% similarity with the 16S rRNA nucleotide sequences of *Streptomyces griseorubens* strain NBRC 12780^T^ (NR 041066; Figure 4A). For verification purposes, we noticed that the pure cultures produced grey aerial mycelia with no distinctive pigment substrate mycelial growth on ISP medium 3 (Figure 4B). The configuration of the spore chains of BCA3 belonged to section spirals and the strain formed open spiral chains consisting of 5–10 spores on the aerial hyphae (Figure 4C). Mature spore chains were also observed using SEM and found to be moderately short with 5–10 spores per chain. Spore surface was spiny with very short spines. Therefore, BCA3 was assigned as *Streptomyces griseorubens* [73,74] Strain UAE2.

Phylogenetic analysis of the 1511-bp 16S rRNA of BCA5 (GenBank accession number MZ026483) showed 100% similarity to *S.* *wuyuanensis* FX61^T^ (NR 118447) *(*Figure S2); while the rest of other *Streptomyces* spp. showed < 99.4% similarity. Typical grey aerial mycelia and yellowish-white (creamy) substrate mycelia were observed when BCA5 was cultivated (Figure S2). BCA5 showed long spiral chains (section: spirals) and smooth-surfaced and non-motile spores (Figure S2). This suggests that BCA5 (#45) could be most probably recognized as *Streptomyces* *wuyuanensis* [75] strain UAE1.

### 3.7. Additive Effect of S. griseorubens UAE2 on N. dimidiatum under Container Nursery Conditions

**Figure 5:**
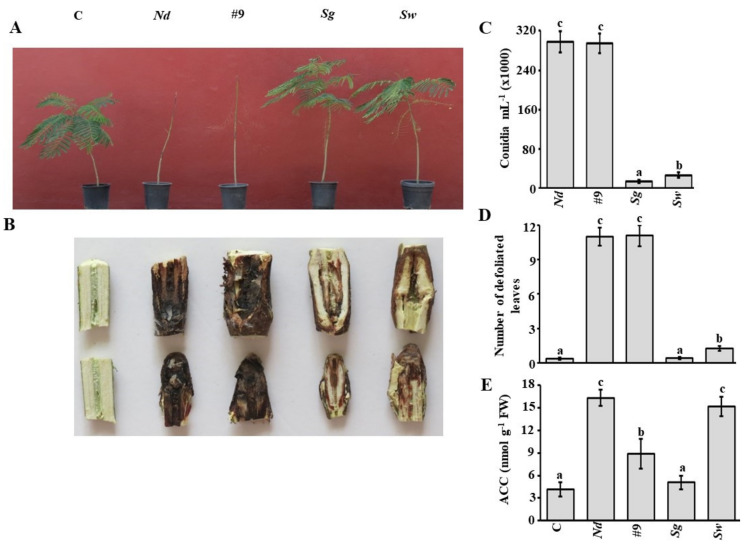
Effect of biocontrol application on inoculated-royal poinciana inoculated with *Neoscytalidium dimidiatum*. Effect of the preventive (**A**) BCA treatment; and (**B**) symptoms of inoculated regions with *N. dimidiatum*. Number of (**C**) conidia after the pathogen recovery from affected stem tissues; (**D**) defoliated leaves; and (**E**) endogenous contents of ACC in stem tissues of inoculated royal poinciana seedlings after BCA treatment after 10 wpt growing under container nursery conditions. In (**C**–**E**), values with different letters are significantly different from each other at *p* < 0.05. Bars represent standard errors. C, non-inoculated control seedlings, *Nd*, seedlings inoculated with *N. dimidiatum* only; #9/*Sg*/*Sw*, seedlings inoculated with the individual isolate two weeks prior to *N. dimidiatum* inoculation. #9, non-antifungal-, non-CWDE- and ACCD-producing isolate #9 (control); *Sg,* ACCD-producing *Streptomyces griseorubens* UAE2 (BCA3; isolate #21); *Sw,* ACCD-non-producing *Streptomyces wuyuanensis* UAE1 (BCA5; isolate #45); FW, fresh weight; ACC, 1-aminocyclopropane-1-carboxylic; ACCD, ACC deaminase; *Nd, N. dimidiatum*; BCA, biological control agent; wpt, weeks post treatment.

In the container nursery experiment, typical symptoms of stem canker disease, such as falling leaves and discolored stems, were observed on seedlings inoculated with *N. dimidiatum* (*Nd*) until they became completely bare by nine wpi (Figure 5A). Clearly, longitudinal necrosis on woods was spotted in diseased plants (Figure 5B). In addition, we determined the effect of the ACCD-producing *S. griseorubens* UAE2 (*Sg*+*Nd*) and non-ACCD-producing *S. wuyuanensis* UAE1 (*Sw*+*Nd*) on royal poinciana two weeks prior to inoculation with *N. dimidiatum* (preventive treatment). When seedlings were treated with isolate #9, we did not notice any difference in disease symptoms with seedlings inoculated with the pathogen alone (Figure 5A,B). This suggests that isolate #9, lacking biological control properties, did not have the efficacy to manage stem canker disease albeit producing ACCD. Individual treatments of either *Sg* (BCA3) or *Sw* (BCA5) suppressed stem canker disease in varying degrees (Figure 5A). According to the disease symptoms on seedlings, application of *S. griseorubens* was more effective on decreasing pathogen invasion success than of *S. wuyuanensis* treatment. This was evident from the minimal damage of necrotic tissues seen on seedlings caused by *Sg*+*Nd* compared to *Sw*+*Nd* treatment (Figure 5B).

We confirmed our results by checking the conidial counts recovered and the number of defoliated leaves from diseased plants. The *Sg*+*Nd* treatment caused a greater reduction by about two-fold in the number of conidia, followed by *Sw*+*Nd* treatment (Figure 5C). We found that *S. wuyuanensis* applied before *N. dimidiatum* on plants was significantly (*p* < 0.05) effective compared to isolate #9 applied prior to *N. dimidiatum*. This was clearly demonstrated in the conidial counts which were lesser in *Sw*+*Nd* than in diseased seedlings inoculated with isolate #9. The number of conidia recovered from plants treated with isolate #9 treatment was comparable to that of *Nd* alone.

The number of falling leaves in seedlings inoculated with *N. dimidiatum* alone or isolate #9 significantly (*p* < 0.05) increased compared to any BCA-inoculated or non-inoculated seedlings at 10 wpt (Figure 5D). Royal poinciana seedlings treated with BCA3 or BCA5 before *N. dimidiatum* infection (*Sg*+*Nd* or *Sw*+*Nd*, respectively) significantly (*p* < 0.05) reduced number of defoliated leaves, thus *Sg*+*Nd* treatment was comparable to the corresponding control treatment (no pathogen infection). Although there was relative superiority of *S. griseorubens* over *S. wuyuanensis* in our *in vivo* assays on diseased-royal poinciana seedlings in the greenhouse/nursery, both *Streptomyces* spp. did not show significant (*p >* 0.05) difference in their antagonistic activities against *N. dimidiatum in vitro* (Table 1).

Infection of *N. dimidiatum* alone or combined with *Sw* significantly (*p* < 0.05) increased ACC levels *in planta* (Figure 5E). Seedlings amended with *Sg*, on the other hand, had significantly (*p* < 0.05) lower ACC levels in plant tissues than in all other treatments, and was comparable to no pathogen treatment. This suggests that this ACCD-producing isolate (*S. griseorubens*) could possibly improve its performance for better plant health. Taken together, we found that the presence of *S.* *wuyuanensis* mimicked disease progression, but additive inhibitory effects exhibited on *N. dimidiatum* in the existence of *S. griseorubens*. This suggests that the latter *Streptomyces* spp. not only performs as a strong BCA, but also possesses a powerful stress reliever to manage stem canker disease on royal poinciana.

## 4. Discussion

In our screening for BCAs to limit the growth of *N. dimidiatum*, we obtained 47 actinobacterial (38 SA and 9 NSA) isolates from the rhizosphere soils of healthy royal poinciana trees in the UAE without the use of *Streptomyces* phages or dry heat methods. Isolates of *Streptomyces* species were the most dominant; whereas NSA strains have been rarely isolated and are generally less abundant in soils [24,76]. To increase the number and the diversity of NSA from royal poinciana rhizosphere soils, polyvalent *Streptomyces* phages and dry heat approaches were used in the current study. These methods resulted in obtaining 44 additional NSA isolates. *Streptomyces* phages can eliminate *Streptomyces* colonies from isolation plates [45]. Physical treatments (e.g., dry heat method) have been commonly applied for the isolation of NSA genera including *Actinoplanes,* *Rhodococcus, Nocardia, Actinomadura*, *Micromonospora, Microbispora*, *Streptosporangium,* and others [77,78]. Isolates of NSA have previously been reported to manage many diseases including cavity spot disease of carrots [25], damping-off of cucumber [23] and dieback disease on mango [14]. In the current study, none of NSA isolates were however able to control stem canker disease. Our findings were in agreement with other studies that SA are the most predominant group of soil actinobacteria and are a “treasure house” of secondary and bioactive metabolites [20].

For efficient selection of BCAs [79], the first step was to identify actinobacterial isolates according to their *in vitro* capabilities to produce both diffusible antifungal metabolites and CWDEs against *N. dimidiatum*. Our data confirmed previous reports about the importance of actinobacteria as potential BCAs against phytopathogens [10,14]. Due to the high number of isolates obtained from the primary *in vitro* screening assays, potential BCA antagonists to *N. dimidiatum* were further chosen from the mature apple fruit bioassay. Such *in vivo* antagonistic bioassays of BCA pairing with *Pythium coloratum* on carrot, *Lasiodiplodia theobromae* on mango and *N. dimidiatum* on apple have predicted the efficacy of BCA on seedlings against phytopathogens in greenhouse and field studies [7,14,25,28]. Because our aim was to find a BCA candidate with multiple BCA characteristics including ACCD production, only two isolates were chosen. Thus, BCA3 (isolate #21) was further characterized as the most promising strain due to the highly diffusible antifungal metabolites, CWDEs and ACCD produced in addition to other biochemical substances produced (e.g., volatiles compounds and siderophores). Based on 16S rRNA similarity with other *Streptomyces* species, BCA3 was identified as *S. griseorubens* UAE2. Except of ACCD production, all *in vitro* properties of *S. wuyuanensis* UAE1 (BCA5; isolate #45) were comparable to those found in *S. griseorubens* UAE2 (Table 1).

To determine if ACCD activity had an additional effect, the two indigenous BCA candidates, *S. griseorubens* UAE2 (ACCD-producer) and *S. wuyuanensis* UAE1 (non-ACCD producer) were evaluated for their effectiveness against stem canker disease on royal poinciana under greenhouse (Figure 2) and container nursery conditions (Figure 5). Previously, *S. griseorubens* E44G and MPT42 strains isolated from soils and stems of *Litsea cubeba* have been identified as strong antagonists possessing broad antifungal activities [80,81]. Zhang et al. [75] have isolated *S. wuyuanensis* FX61^T^ from a saline sample collected from Wuyuan, China, thus the current study is the first to report a strain of *S. wuyuanensis* potent to be developed as a BCA to minimize the utilization of hazardous chemicals in plant disease management.

In the present study, both isolates enhanced resistance in royal poinciana against *N. dimidiatum*, thus the ACCD-producing *S. griseorubens* UAE2 was relatively superior as a BCA (Figure 2 and Figure 5). Similar to the preventive application of *S. wuyuanensis* on royal poinciana diseased plants, pre-inoculation of mango plants with *Streptomyces samsunensis* resulted in high disease protection in mango against *L. theobromae* [14]. The antifungal action of *S. wuyuanensis* and *S. samsunensis* was related to antibiosis and production of CWDEs and siderophores. A mixture of SA and NSA isolates possessing multiple modes of action has been reported effective in controlling cucumber rot diseases caused by *Pythium aphanidermatum* [82]. Recently, Al Raish et al. [28] have screened SA isolates retaining one or more modes of action (antibiosis and/or production of CWDEs), thus the best results have been obtained when *S. antibioticus* UAE1 (a BCA with multiple mechanisms) was applied to manage the disease caused by *N. dimidiatum* compared to *Streptomyces* spp. possessing a single mechanism.

One strategy to reduce ET levels was to inoculate plants with an actinobacterium active in ACCD production. In comparison to *S. wuyuanensis*, *S. griseorubens* showed not only synergistic mechanisms of antagonism, but also had additive effect of suppression on stem canker disease in the nursery experiments (Figure 5). This could be attributed mainly to the secretion of ACCD by *S. griseorubens* to provide systemic suppression of *N. dimidiatum* for the benefit of the host plant. Isolate #9 did not have this privilege in suppressing the disease on royal poinciana, although it was considered an ACCD producer. We argue that the additive effect of ACCD activity should be accompanied with other biological control properties (e.g., production of diffusible antifungal metabolites and CWDEs) in BCAs.

Even though several studies have evaluated bacterial ACCD activity in response to abiotic stresses [83,84,85,86], others have reported that plants inoculated with non-actinobacterial strains containing ACCD can make plants resistant to various phytopathogens [35,36,37]. For example, rhizosphere bacteria producing ACCD can act as promising BCAs against pathogenic strains of *Agrobacterium* in tomato plants [87]. Furthermore, Yim et al. [38] demonstrated that tomato plants treated with the BCA *Methylobacterium* sp., which was capable of producing ACCD and inoculated with *Ralstonia solanacearum*, significantly reduced wilt symptoms and lowered ET emission under greenhouse conditions compared to the control treatment. In the current study, we claim that *S. griseorubens* is more efficient BCA than *S. wuyuanensis* on royal poinciana trees to control *N. dimidiatum*. This is the first study to report an actinobacterial isolate producing ACCD as a potential BCA for a tree disease, thus suggesting that the inoculation of trees grown in the nursery under natural conditions might constitute a novel strategy to obtain *N. dimidiatum* resistant royal poinciana trees.

It is worth mentioning that actinobacteria are as well-adapted to extreme heat and local dry soils [88] as those found in the UAE. This could potentially implement actinobacterial BCAs, such as *S. griseorubens* UAE2, as a safe and natural enemy to *N. dimidiatum* in IDM programs. This study is the first report of a biocontrol of stem canker on royal poinciana caused by *N. dimidiatum* managed by applying a *Streptomyces* species producing ACCD and the second report of employment of *Streptomyces* spp. as BCAs against *N. dimidiatum* in the UAE [28]. Field studies on naturally infested plants are at the top of our list of priorities for fine-tuning farming schemes aimed at pathogen prevention.

## Figures and Tables

**Table 1:** Antagonism against *Neoscytalidium* *dimidiatum* and production of ACCD by 20 SA and NSA isolates.

Species	Isolate	*In Vitro*		*In Vivo*	*In Vitro*
		Inhibition Zone Diameter (mm) ^a^	Clearing Zone Diameter (mm) ^b^	Lesion Diameter (mm) ^c^	ACCD Activity (Nmol α-Ketobutyrate/mg Protein/h) ^d^
*N. dimidiatum* (negative control)		0.00	0.00	32.34 ± 0.29 *e*	0.00 *a*
#9 (positive control)		0.00	0.00	31.98 ± 0.32 *e*	406.06 ± 13.12 *e*
*Streptomyces*	#4 (BCA1)	47.80 ± 2.22 *e*	54.23 ± 1.83 *d*	0.34 ± 0.04 *a*	41.88 ± 2.62 *b*
	#8	35.10 ± 0.24 *c*	26.32 ± 0.18 *a*	14.34 ± 0.03 *c*	90.08 ± 4.04 *c*
	#10 (BCA2)	45.26 ± 2.90 *d*	53.73 ± 1.88 *d*	0.16 ± 0.02 *a*	0.00 *a*
	#19	25.45 ± 0.15 *a*	26.34 ± 0.52 *a*	25.16 ± 1.10 *a*	81.18 ± 3.24 *c*
	#21 (BCA3)	48.01 ± 2.07 *e*	55.64 ± 1.78 *d,e*	0.18 ± 0.04 *a*	527.42 ± 15.84 *f*
	#23 (BCA4)	45.94 ± 3.06 *d*	54.87 ± 1.62 *de*	0.86 ± 0.05 *a*	0.00 *a*
	#29	45.76 ± 3.46 *d*	56.67 ± 2.35 *e*	26.36 ± 1.67 *d*	0.00 *a*
	#32	46.98 ± 0.88 *de*	27.06 ± 0.08 *a*	7.40 ± 0.05 *b*	0.00 *a*
	#37	46.78 ± 0.56 *d*	37.22 ± 0.82 *b*	8.16 ± 0.22 *b*	80.88 ± 4.26 *c*
	#44	47.30 ± 2.32 *e*	60.16 ± 3.04 *f*	32.65 ± 1.34	25.40 ± 2.00 *b*
	#45 (BCA5)	48.66 ± 0.26 *e*	55.24 ± 1.08 *d,e*	0.14 ± 0.02 *a*	0.00 *a*
	#53	46.68 ± 2.54 *d*	56.52 ± 0.56 *e*	31.17 ± 0.61 *e*	0.00 *a*
*Micromonospora*	#7	46.17 ± 3.52 *d*	52.84 ± 2.3 *d*	31.18 ± 0.18 *e*	30.50 ± 2.08 *b*
	#42 (BCA6)	46.62 ± 1.46 *d*	55.35 ± 2.18 *d,e*	0.10 ± 0.01 *a*	0.00 *a*
*Actinomadura*	#14	26.16 ± 0.17 *a*	44.82 ± 1.12 *c*	15.15 ± 0.34 *c*	26.54 ± 1.98 *b*
*Dactylosporangium*	#38 (BCA7)	46.46 ± 2.26 *d*	54.84 ± 2.14 *d,e*	0.25 ± 0.05 *a*	280.18 ± 10.60 *d*
*Streptosporangium*	#41	35.36 ± 0.92 *c*	56.86 ± 1.88 *e*	6.68 ± 0.04 *b*	0.00 *a*
*Microbispora*	#48	33.81 ± 1.25 *c*	35.83 ± 3.46 *b*	13.18 ± 0.06 *c*	28.70 ± 1.66 *b*
*Microtetraspora*	#25	28.82 ± 0.76 *b*	27.66 ± 0.18 *a*	32.02 ± 0.10 *e*	439.68 ± 13.16 *e*
*Actinoplanes*	#56	47.78 ± 0.22 *e*	26.06 ± 0.12 *a*	8.08 ± 1.02 *b*	0.00 *a*

^a^ Production of diffusible antifungal metabolites active against *N. dimidiatum* using the cut-plug method. ^b^ Production of CWDEs on mycelial fragment agar. ^c^ Effect of the antagonistic BCA on *N. dimidiatum* using the *in vivo* apple fruit bioassay. ^d^ Production of ACCD in DF medium amended with ACC after five days of incubation at 28 ± 2 °C. Values are means ± SE of eight replicates for *in vitro* experiments for production of diffusible antifungal metabolites and CWDEs; eight replicates for *in vitro* ACCD production, and five replicates for *in vivo* fruit bioassay. Values within each column, followed by the same letter are not significantly (*p* > 0.05) different according to Duncan’s multiple range test. Isolate #9 is a non-antifungal-, non-CWDE- and ACCD-producing positive control. Isolates #21, #45 and 9 represent *Streptomyces griseorubens* (BCA3), *Streptomyces wuyuanensis* (BCA5) and *Streptomyces* sp.; respectively. BCA, biological control agent; SA, streptomycete actinobacteria; NSA, non-streptomycete actinobacteria; ACC, 1-aminocyclopropane-1-carboxylic acid; ACCD, ACC deaminase; CWDEs, cell-wall-degrading enzymes; DF, Dworkin and Foster’s salts minimal broth medium.

**Table 2:** Effects of the crude culture filtrate of the two BCA candidates obtained from FMEB and CCB on the mycelial, conidial and germ tube characteristics of *Neoscytalidium* *dimidiatum*.

Media	BCA	Culture Filtrate (%)	Colony Diameter (mm)	Mycelial Dry Weight (g)	Conidia Germination (%)	Germ Tube Length (µm)
FMEB	BCA3	0	97.94 ± 0.64 *c*	76.95 ± 3.04 *c*	87.62 ± 1.16 *c*	48.45 ± 2.40 *c*
		50	12.43 ± 1.16 *b*	6.28 ± 1.56 *b*	15.96 ± 2.20 *b*	14.24 ± 2.03 *b*
		100	0.00 ± 0.00 *a*	0.00 ± 0.00 *a*	0.94 ± 0.26 *a*	2.04 ± 0.32 *a*
	BCA5	0	96.78 ± 0.32 *c*	79.54 ± 2.74 *c*	85.82 ± 1.72 *c*	55.78 ± 3.00 *c*
		50	10.86 ± 1.04 *b*	4.96 ± 1.65 *b*	17.78 ± 1.56 *b*	13.34 ± 1.62 *b*
		100	0.00 ± 0.00 *a*	0.00 ± 0.00 *a*	0.74 ± 0.32 *a*	1.43 ± 0.29 *a*
CCB	BCA3	0	96.78 ± 1.12 *c*	78.22 ± 4.00 *c*	91.92 ± 1.88 *c*	66.60 ± 2.60 *c*
		50	14.86 ± 1.38 *b*	12.56 ± 0.99 *b*	19.28 ± 1.04 *b*	9.67 ± 1.08 *b*
		100	0.00 ± 0.00 *a*	0.00 ± 0.00 *a*	0.97 ± 0.12 *a*	0.00 ± 0.00 *a*
	BCA5	0	97.12 ± 1.46 *c*	80.92 ± 2.86 *c*	88.62 ± 2.08 *c*	62.99 ± 3.12 *c*
		50	16.92 ± 0.62 *b*	13.10 ± 1.66 *b*	20.67 ± 0.88 *b*	10.32 ± 0.75 *b*
		100	0.00 ± 0.00 *a*	0.00 ± 0.00 *a*	1.82 ± 0.34 *a*	0.00 ± 0.00 *a*

Values are means ± SE of eight replicates. Values with the same letter within a column for each BCA are not significantly (*p* > 0.05) different, according to Duncan’s multiple range test. BCA3 (isolate #21) and BCA5 (#45) represent *Streptomyces griseorubens* and *S. wuyuanensis*; respectively. FMEB, fish meal extract broth; CCB, colloidal chitin broth; BCA, biological control agent.

## Data Availability

All sequences were deposited in Genbank (NCBI; https://www.ncbi.nlm.nih.gov/ accessed on 25 September 2021) with accession numbers MZ021577 for *Streptomyces griseorubens* UAE2 and MZ026483 for *Streptomyces* *wuyuanensis* UAE1.

## Supplementary Materials

The following are available online at https://www.mdpi.com/article/10.3390/jof7110885/s1, Table S1: Comparison on the effect of application of polyvalent *Streptomyces* phages and dry heat techniques on isolation plates of cfu of SA and NSA from rhizosphere soils of royal poinciana, Figure S1: *In vitro* effect of BCA candidates on mycelial growth of *Neoscytalidium dimidiatum*. Inhibition of *N. dimidiatum* mycelial growth by the BCAs using (A) cut-plug; and (B) cup plate methods. In (A,B), the diffusible antifungal metabolite-producing isolate #21 and #45 compared to the diffusible antifungal metabolite-non-producing isolate #9 and control. In (B), wells were inoculated with filter-sterilized fish meal extract broth of isolates #21, #45 or #9. Isolates #21 and #45 represent *Streptomyces griseorubens* (BCA3) and *Streptomyces wuyuanensis* (BCA5); respectively. BCA, biological control agent. Table S2: *In vitro* antagonistic activities of the strongest seven BCA candidates showing antagonism to *Neoscytalidium* *dimidiatum*, Table S3: *In vitro* comparisons of antagonistic activities between the strongest ACCD- producing BCA and the ACCD-non-producing BCA candidates against *Neoscytalidium* *dimidiatum,* Figure S2: Identification of *Streptomyces wuyuanensis* UAE1. (A) The phylogenetic tree showing the relationships between *S. wuyuanensis* UAE1 (BCA5, ACCD-non-producing isolate) (isolate 45; MZ026483; 1511 bp) and other *Streptomyces* spp. based on 16S rRNA sequences. (B) Gray aerial (left) and yellowish-white substrate (right) mycelia growing on ISP3 medium supplemented with yeast extract. (C) Scanning electron micrograph (8500X) of spirales spore chains (red arrow) and smooth-surfaced spores (yellow arrow) of *S. wuyuanensis* UAE1. In (A), numbers at nodes indicate percentage levels of bootstrap support based on a neighbor-joining analysis of 500 resampled datasets. GenBank accession numbers are given in parentheses. BCA, biological control agent.
