# Supplementary material for: Effectiveness of Augmentative Biological Control of Streptomyces griseorubens UAE2 Depends on 1-Aminocyclopropane-1-Carboxylic Acid Deaminase Activity against Neoscytalidium dimidiatum
Source: J Fungi (Basel). 2021 Oct 20;7(11):885. doi: 10.3390/jof7110885 (PMC8618148; doi:10.3390/jof7110885)
Supplement: Supplementary file 1 [file jof-07-00885-s001.zip › jof-1416471-supplementary.pdf]

Supplementary Material

Effectiveness of Augmentative Biological Control of *Streptomyces griseorubens* UAE2 Depends on 1-Aminocyclopropane-1-Carboxylic Acid Deaminase Activity Against *Neoscytalidium dimidiatum*

Bader M. Al Hamad, Seham M. Al Raish, Gaber Ramadan, Esam E. Saeed, Shaikha S.A. Alameri, Salima S. Al Senaani, Synan F. AbuQamar*, Khaled A. El-Tarabily *

* Correspondence: sabuqamar@uaeu.ac.ae (S.AQ.)
ktarabily@uaeu.ac.ae (K.E.-T.)

Table S1. Comparison on the effect of application of polyvalent *Streptomyces* phages and dry heat techniques on isolation plates of cfu of SA and NSA from rhizosphere soils of royal poinciana.

**Actinobacteria**	**Without phage**	**With phage**	**Without heat**	**With heat**
	**$\log_{10}$ cfu g soil^{-1}**			
SA	7.14±0.63 *a*	1.85±0.31*b*	6.88±0.41 *a*	2.81±0.68 *b*
NSA	1.88±0.36 *a*	5.67±0.41 *b*	1.52±0.62 *a*	4.78±0.93 *b*

Values are means ± SE of 8 replicates. For each method, values (within rows) followed by the same letter are not significantly (P>0.05) different according to Duncan's multiple range test. cfu, colony-forming units; SA, streptomycete actinobacteria; NSA, non-streptomycete actinobacteria.

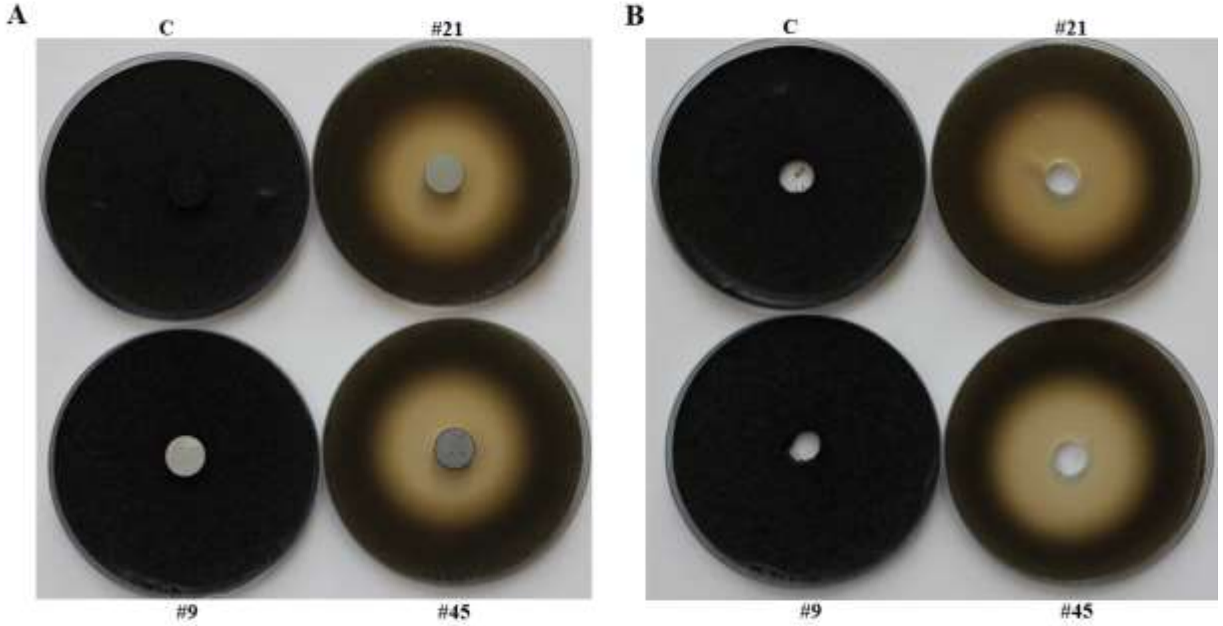

Figure S1. *In vitro* effect of BCA candidates on mycelial growth of *Neoscytalidium dimidiatum*. Inhibition of *N. dimidiatum* mycelial growth by the BCAs using (A) cut-plug; and (B) cup plate methods. In (A & B), the diffusible antifungal metabolite-producing isolate #21 and #45 compared to the diffusible antifungal metabolite-non-producing isolate #9 and control. In (B), wells were inoculated with filter-sterilized fish meal extract broth of isolates #21, #45 or #9. Isolates #21 and #45 represent *Streptomyces griseorubens* (BCA3) and *Streptomyces wuyuanensis* (BCA5); respectively. BCA, biological control agent.

Table S2. *In vitro* antagonistic activities of the strongest seven BCA candidates showing antagonism to *Neoscytalidium dimidiatum.*

Activities	Isolate						
	+ACCD			-ACCD			
	BCA1	BCA3	BCA7	BCA2	BCA4	BCA5	BCA6
Production of volatile compounds [a]	**-**	+	+	+	+	**+**	-
Production of siderophores [b]	+	+	-	-	+	+	+
Production of hydrogen cyanide [c]	-	-	-	-	-	-	-

[a] + = fungicidal effect; - = no fungicidal effect.
[b] + = produced; - = not produced.
[c] - = not produced.
BCA1, BCA3 (*Streptomyces griseorubens*) and BCA7 represent isolates #4, #21 and #38, respectively. BCA2, BCA4, BCA5 and BCA6 represent isolates #10, #23, #45 (*Streptomyces wuyuanensis*) and #42, respectively. BCA, biological control agent.

Table S3. *In vitro* comparisons of antagonistic activities between the strongest ACCD- producing BCA and the ACCD-non-producing BCA candidates against *Neoscytalidium dimidiatum*.

Activities	Isolate	
	BCA3	BCA5
**Production of diffusible metabolites using**		
cup plate (diameter of inhibition zone in mm)	63.90±3.24 *a*	65.68±2.34 *a*
**Chitinase from**		
colloidal chitin (U ml^{-1}) [a]	6.06±1.22 *a*	5.84±1.14 *a*
N. dimidiatum cell-wall (U ml^{-1}) [a]	4.16±0.62 *a*	4.02±1.05 *a*
**ß-1,3-glucanase from**		
laminarin (U ml^{-1}) [b]	5.08±1.10 *a*	4.94±1.20 *a*
N. dimidiatum cell-wall (U ml^{-1}) [b]	2.00±0.38 *a*	1.97±0.16 *a*

[a] A unit of chitinase was expressed as the amount of the enzyme that released 1 μmol of N-acetyl-D-glucosamine mg^{-1} protein h^{-1}. This indicates that BCA3 and BCA5 produced high levels of chitinase.
[b] A unit of ß-1,3-glucanase was expressed as the amount of the enzyme that released 1 μmol of glucose mg^{-1} protein h^{-1}. This indicates that BCA3 and BCA5 produced high levels of ß-1,3-glucanase.
Values are means±SE from eight replicates. Values with the same letter within a row are not significantly (P>0.05) different according to Duncan's multiple range test.
BCA3 (ACCD-producing isolate #21) and BCA5 (ACCD-non-producing isolate #45) represent *Streptomyces griseorubens* and *Streptomyces wuyuanensis*; respectively.
FMEA, fish meal extract agar; CCA, colloidal chitin agar.
BCA, biological control agent.

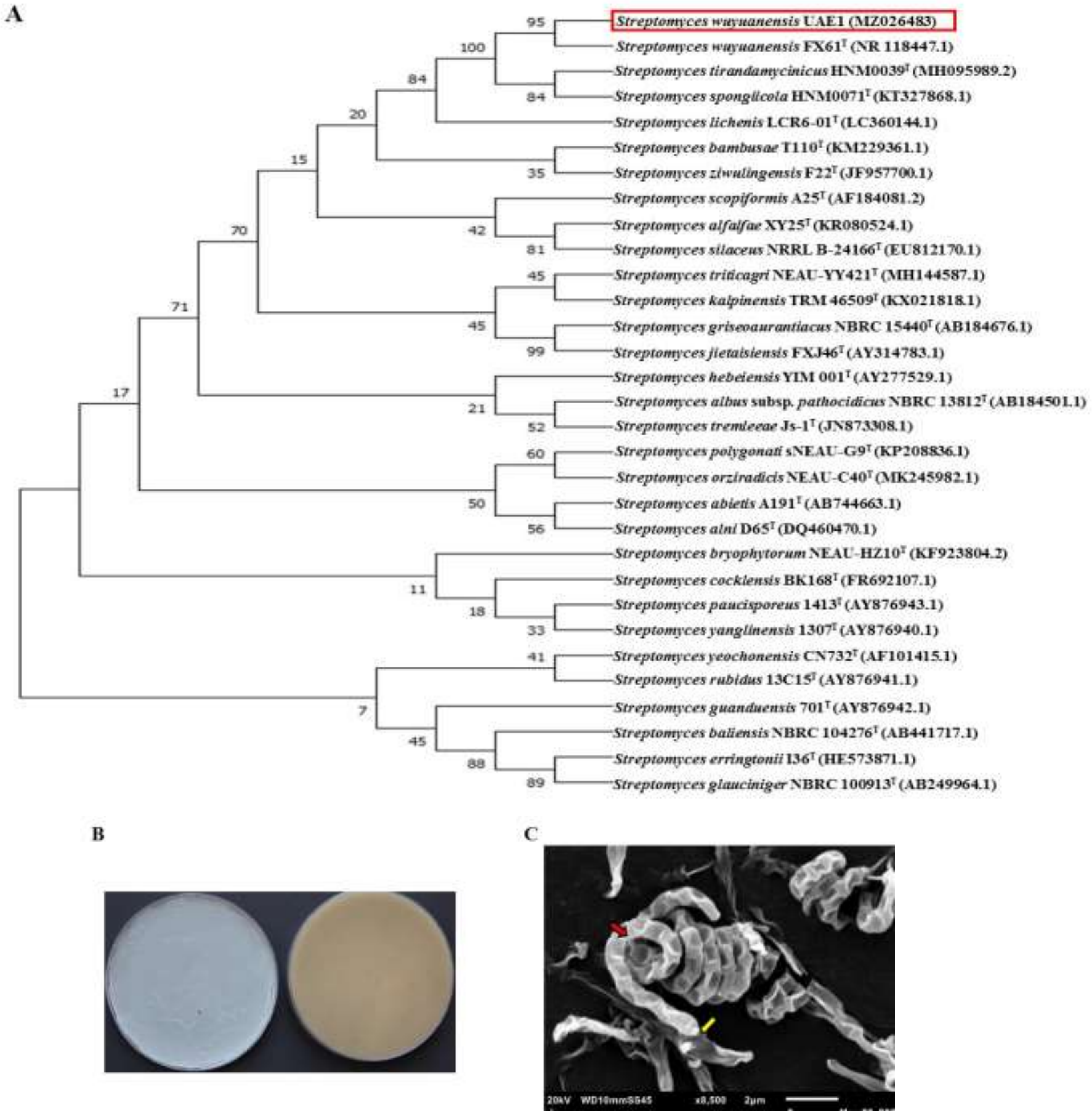

Figure S2. Identification of *Streptomyces wuyuanensis* UAE1. (A) The phylogenetic tree showing the relationships between *S. wuyuanensis* UAE1 (BCA5, ACCD-non-producing isolate) (isolate 45; MZ026483; 1,511 bp) and other *Streptomyces* spp. based on 16S rRNA sequences. (B) Gray aerial (left) and yellowish-white substrate (right) mycelia growing on ISP3 medium supplemented with yeast extract. (C) Scanning electron micrograph (8500X) of spirales spore chains (red arrow) and smooth-surfaced spores (yellow arrow) of *S. wuyuanensis* UAE1. In (A), numbers at nodes indicate percentage levels of bootstrap support based on a neighbor-joining analysis of 500 resampled datasets. GenBank accession numbers are given in parentheses. BCA, biological control agent.
